# RESPIRE: The National Institute for Health Research's (NIHR) Global Respiratory Health Unit

**DOI:** 10.7189/jogh.08.020101

**Published:** 2018-12

**Authors:** Aziz Sheikh, Harry Campbell, Dominique Balharry, Abdullah H. Baqui, Debby Bogaert, Kathrin Cresswell, Steve Cunningham, David Dockerell, Shams El Arifeen, Monica Fletcher, Liz Grant, Sazlina Shariff Ghazali, Monsur Habib, Tabish Hazir, Rita Isaac, Sanjay Juvekar, Ee Ming Khoo, Brian McKinstry, Andrew D Morris, Harish Nair, John Norrie, Bright I Nwaru, Hilary Pinnock, Dave Robertson, Samir Saha, Sundeep Salvi, Jürgen Schwarze, Colin Simpson, Devi Sridhar, Andrew Stoddart, David Weller, Moira Whyte, Allison Worth, Siân Williams, Osman Yusuf, Alimuddin Zumla, Igor Rudan, on behalf of the RESPIRE Collaboration

**Affiliations:** 1 Usher Institute, The University of Edinburgh, Edinburgh, Edinburgh, UK; 2 Projahnmo Research Foundation, Bangladesh, Dhaka, Bangladesh; 3 MRC Centre for Inflammation Research, The University of Edinburgh, Edinburgh, UK; 4 Child Life and Health, The University of Edinburgh, UK; 5 Maternal and Child Health Division (MCHD), International Centre for Diarrhoeal Disease Research (icddr,b), Dhaka, Bangladesh; 6 Global Health Academy, The University of Edinburgh, Edinburgh, UK; 7 Department of Family Medicine, University Putra Malaysia, Selangor, Malaysia; 8 Bangladesh Primary Care Respiratory Society, Khulna, Bangladesh; 9 Pakistan Institute of Medical Sciences, Islamabad, Pakistan; 10 Maternal Neonatal and Child Health Research Network (MNCHRN), Pakistan; 11 Rural Unit for Health and Social Affairs, Christian Medical College, Vellore, India; 12 Vadu Rural Health Program, King Edward Memorial Hospital Research Centre (KEMHRC) Pune, India; 13 Department of Primary Care Medicine, Faculty of Medicine, University of Malaya, Malaysia; 14 College of Science and Engineering, The University of Edinburgh, Edinburgh, UK; 15 Child Health Research Foundation, Dhaka, Bangladesh; 16 Chest Research Foundation, Pune, India; 17 Edinburgh Clinical Research Facility, The University of Edinburgh, Edinburgh, UK; 18 International Primary Care Respiratory Group, Edinburgh, UK; 19 Allergy and Asthma Institute, Islamabad, Pakistan; 20 Division of Infection and Immunity, University College London, London, UK; 21 NIHR Biomedical Research Centre, UCL Hospitals NHS Foundation Trust, London, UK; 22 Faculty of Health, Victoria University of Wellington, New Zealand; 23 Krefting Research Centre, Institute Of Medicine, Gothenburg, Gothenburg, Sweden

## CONTEXTUAL CONSIDERATIONS: THE NEED FOR RESPIRE

The National Institute of Health Research (NIHR) Global Health Research Unit on Respiratory Health (RESPIRE) aims to reduce morbidity and mortality from respiratory diseases globally, with an initial focus on South Asia where respiratory conditions account for around one in five of all deaths. This geographically focused approach allows RESPIRE to develop a regional network of excellence in four South Asian countries, namely Bangladesh, India, Malaysia and Pakistan, though over time the intention is to extend the Unit’s activities. Interested colleagues from other countries are invited to contact RESPIRE via the website (www.ed.ac.uk/usher/respire) which gives more information on the work of the Unit, and provides contact details on how to get involved and support RESPIRE’s goals in other countries.

In the 2017 Global Burden of Disease (GBD) study, lower respiratory tract infections (LRTI), chronic obstructive pulmonary disease (COPD), tuberculosis (TB) and lung cancer were all ranked among the top 12 medical conditions (out of nearly 300) in terms of global premature mortality measured as Years of Life Lost (YLL) [1-5]. COPD was ranked 6^th^ (female) and 9^th^ (male) in terms of disability, measured as Years of Life with Disability (YLD) [1-5]. In fact, in 2016, COPD was the 3^rd^ highest cause of death globally, claiming 3 million lives, while lung cancer (along with trachea and bronchial cancers) was the 6th highest [6]. LRTIs were ranked 4^th^, making them the most deadly set of communicable diseases, causing 3 million deaths worldwide [6]. In 2017, in South Asia alone, just five respiratory conditions accounted for 14.4% of all Disability-Adjusted Life Years (DALYs; LRTIs, 4.9%; COPD, 4.6%; TB, 3.0%; asthma, 1.4%; lung cancer, 0.5% of total DALYs) [7]. Furthermore, awareness of their relative importance is low and these conditions have not attracted the attention and priority they deserve from health systems, policy makers and funding agencies nationally, within the South Asia region, or indeed globally [5,8].

Despite this substantial morbidity and mortality from both communicable and non-communicable respiratory conditions in low- and middle-income countries (LMICs), they are currently relatively neglected by global research funders. Much of this ill-health is preventable through bridging the ‘second translational gap’ [9]. In the target LMICs, this necessitates a detailed appreciation of local challenges – for example, social stigma associated with diagnoses of asthma, limited appreciation of risk factors (eg, smoking/biomass fuel exposure) and the role of self-management, the use of alternative therapies, high clinician workloads and limited continuity of care. Using these to develop innovative tools/solutions involves mobilisation of local assets aligned with global opportunities.

The RESPIRE Collaboration recognises the value of achieving impact by adapting and/or culturally tailoring interventions that have already been found to be effective in other populations. RESPIRE has launched implementation studies of adapted interventions in the context of acute LRTIs and chronic respiratory disorders. In the longer-term, they will be complemented by the development of novel interventions for areas where there is no available evidence from other contexts through initiating relevant underpinning work leading to definitive trials.

## AIMS AND STRATEGIC GOALS OF RESPIRE

RESPIRE is dedicated to achieving a step-change in national and regional infrastructures and capacity in applied respiratory research. The specific aims are to develop into a world-leading Unit that will: (i) Map and collate continuing and emerging respiratory challenges; (ii) Prioritise existing evidence-based interventions that have the potential to be adapted to reduce mortality and morbidity in LMICs; (iii) Support local adaptation and tailoring of interventions for deployment in low-resource environments and catalyse developmental work in areas of unmet need; (iv) Support local implementation efforts and evaluation of programmes of work; and (v) Identify the best delivery mechanisms for long-term delivery and scaling-up.

To achieve this, RESPIRE focuses on two programmes of research and three underpinning infrastructures or platforms. The first programme addresses the continuing challenge posed by acute infections such as pneumonia and respiratory syncytial virus (RSV), while the second programme addresses long-term non-communicable diseases – in particular, asthma, COPD and lung cancer. These two research programmes are supported by an underpinning infrastructure of three cross-cutting platforms: (i) Stakeholder engagement & governance (to work with partners in engaging a diverse multi-sectoral group in support of RESPIRE activities); (ii) Training & capacity development (to increase the number of health professionals and researchers who are trained to undertake high quality research, including online courses, distance learning masters, PhD training, and training in research leadership and promotion); and (iii) Methodology & data science (ensuring appropriate study designs are adopted and developing plans to maximise the uses that research data can be put to in safe and secure ways).

Coordinated from The University of Edinburgh, RESPIRE brings together a collaborative group of equal partners from the four countries in South Asia. Capacity and capability is being built in these countries to undertake research that is robust and widely relevant to progress the global respiratory health agenda, whilst also being responsive to each country’s particular context and priorities. A specific focus is to address the problems of the poorest populations in these countries, wherever possible. RESPIRE also looks for up-to-date scientific evidence to find ways to reduce deaths and illness caused by these respiratory conditions.

RESPIRE aims to introduce tailored solutions – such as supporting medical doctors and other health professionals or community workers to deliver care through mobile phones (“mHealth”), and study the impact these have. For problems where there is no relevant up-to-date scientific evidence on which to draw, the RESPIRE Unit searches for innovative strategies and interventions to reduce mortality and morbidity secondary to respiratory diseases using experimental study designs to find out if the new solutions are effective and represent good value for money. For interventions meeting these criteria, RESPIRE is working with partner organisations in South Asian countries towards sustainability and scalability of work done, to ensure that they can be provided in the longer term and extended to other areas.

Initially, RESPIRE is promoting shared understanding and developing effective, reciprocal and ethical working relationships; agreeing local research priorities and commencing adaptation projects in both research programmes; identifying research gaps that require more underpinning work; and establishing working groups to develop new grant applications. In the medium-term, the Unit aims to increase capacity and capability in undertaking applied implementation research; to submit collaborative follow-on grants; and to develop sustainability plans. Over the longer-term, RESPIRE aims to maximise the local, regional and global impact from the new knowledge and experience generated by its programmes; release joint publications in top-tier journals; communicate findings to professionals, policymakers and the general population; apply learning to embrace new clinical contexts and expand partnerships; and secure follow-on grants to sustain RESPIRE activities into the future, in order to reduce morbidity and mortality linked to respiratory disorders.

## National and international collaborations

RESPIRE also works with other countries across Asia and Africa, with a vision of scaling-up efforts in due course. RESPIRE investigators currently lead global research networks on respiratory conditions, which include more than 50 research groups, forming enduring and trusting relationships leading to data sharing, co-authorship of publications and joint meetings with high levels of LMIC participation, such as Respiratory Syncytial Virus Global Epidemiology Network (RSV GEN) [10].

RESPIRE maintains strong relationships with The World Bank, United Nations International Children's Emergency Fund (UNICEF), United States Agency for International Development (USAID), Global Alliance against Chronic Diseases, The Joint United Nations Programme on HIV and AIDS (UNAIDS) and The United Nations Population Fund (UNFPA). Its investigators serve on The World Health Organization (WHO) Expert Groups for Air pollution, Childhood pneumonia, Influenza and RSV. RESPIRE also enjoys strong relationships with charitable sector partners, such as Asthma UK, the British Lung Foundation, Bill and Melinda Gates Foundation, Save the Children, The Wellcome Trust as well as professional communities such as the International Primary Care Respiratory Group (IPCRG). Its investigators are Editors-in-Chief of “Nature Partner Journals: Primary Care Respiratory Medicine” and “Journal of Global Health”. IPCRG’s network is facilitating dissemination to other LMICs.

RESPIRE investigators have close working relationships with the Respiratory Industry Group of the Association of British Pharmaceutical Industries (ABPI) and TATA communications, the latter providing mobile connectivity in over 600 networks, including all partner countries.

## TWO PROGRAMMES OF RESEARCH

RESPIRE harbours research programmes in: (i) Acute LRTIs – including bronchiolitis, bronchitis and pneumonia; and (ii) Chronic respiratory disorders – including asthma, COPD, and lung cancer. Specific local projects have been prioritised in association with LMIC partners. RESPIRE is also open to playing a responsive role in the context of pandemics (eg, influenza), antimicrobial stewardship, and data science/security through, for example, shifting resources/staff, to convene expert working groups.

By focusing on high prevalence and high burden, neglected conditions that affect the poorest populations, RESPIRE has identified areas of likely growth. There are opportunities to expand to other respiratory priorities, such as air quality, occupational lung disorders and respiratory co-morbidities.

### Programme 1: Acute lower respiratory tract infectious disorders

RESPIRE programme 1 brings together some of the world leaders in paediatric LRTI research both from the RESPIRE partner countries in South Asia as well as in the UK. The Unit aims to involve other leading international leaders from outwith RESPIRE with significant expertise on specific research questions to collaborate and advise on specific projects. This programme therefore aims to: (i) initiate projects to assess scaling-up and deliverability of affordable and locally adaptable interventions with proven effectiveness against priority concerns relating to childhood acute LRTI in the four RESPIRE countries; (ii) conduct multi-country implementation and experimental studies to inform development of new patient management guidelines that can feed into evidence base for the WHO; (iii) develop new interventions or techniques through small scale projects to serve as proofs of concept for later larger scale trials against complex intractable clinical challenges relating to management of paediatric acute LRTIs.

### Programme 2: Chronic respiratory disorders

There are four main objectives within Programme 2 which are addressed by a range of research projects and PhD studentships across all of the partner countries: (i) to identify and assess the burden of chronic respiratory disease (CRD) in the four partner countries; (ii) to develop and evaluate interventions to improve the management of COPD (and other CRD); (iii) to map the seasonal and geographic allergen load and impact of triggers for asthma; and (iv) to develop tailored approaches to providing supported self-management for asthma.

The primary aim of the major theme on CRDs is to identify the prevalence of COPD and/or asthma, but also other CRDs, amongst adults in Bangladesh, India, Malaysia and Pakistan. RESPIRE also aims to identify risk factors, assess the burden of disease and health care utilisation associated with the development of these conditions. An additional aim is to establish a RESPIRE cohort of people for on-going monitoring and recruitment to future research.

CRDs in low resource settings are neglected and often poorly diagnosed which leads to missed opportunities for early initiation of treatment, and poor patient pathways. A number of projects will develop and evaluate interventions to improve the management of asthma, COPD and other CRDs identified in the RESPIRE prevalence survey, optimising the potential of the RESPIRE cohort.

## THREE UNDERPINNING PLATFORMS

The purpose of RESPIRE platforms is to improve capacity to work with and mobilise national and international partners who can improve the outcomes of the research in terms of its appropriateness, its impact on health systems and individuals and its sustainability. Capacity and capability building by training and development is key to increasing the number of health professionals and researchers who are trained to undertake high quality research for the future. Finally, methodological rigour and world-leading data science expertise is harnessed to maximise the uses that research data can be put to in safe and secure ways.

### Platform I: Stakeholder engagement and governance

Effective stakeholder engagement requires a context-specific understanding and analysis of stakeholders, and action planning, drawing on the best available evidence. There can also be specific ethical and political challenges in LMICs relating to poverty, equity and the current norms and beliefs about public empowerment that need to be addressed sensitively.

RESPIRE stakeholders include donors, policy makers, media, workforce including professional societies and health care employers, other academic departments, citizens, patients and carers. Stakeholders may be involved throughout the research process, including problem identification, research design and planning, research implementation including recruitment, participatory data collection, analysis and interpretation, knowledge translation and implementation and review and adaptation for future studies [11,12,13].

The main aims are: (i) to agree the purposes of the engagement and who should be engaged and when; (ii) to map stakeholders using standard tools; (iii) to analyse the power and impact of each stakeholder at the key phases of each research project; (iv) to develop feasible engagement action plans based on current relationships, trust and priorities and to include these in research protocols; (v) to identify learning needs in the team and to address these effectively; and (vi) to identify learning needs in the stakeholders and suggest to the RESPIRE’s Unit Management Committee (UMC) how these might be addressed through massive online open courses (MOOCs) or other resources.

At the launch, RESPIRE initiated a process with each country team to undertake stakeholder mapping and analysis as a forerunner to developing stakeholder engagement planning and action. It led to some important discussions between team members about different perspectives and relationships. RESPIRE will continue to raise awareness and promote the importance of stakeholder engagement among the country partners.

Future plans include bolstering the role of Patient and Public Involvement (PPI) in global health research. PPI is increasingly becoming standard practice in UK health research and there is a need to ensure the work of RESPIRE remains relevant to the communities in which it works. In addition to identifying ways to embed PPI within the research, it will also be important to explore the use of more community development approaches to ensure the patient voice is heard.

The academic questions in global governance that complement RESPIRE as a whole are: (i) what is the health system in each of these countries, and how does the current health system respond to respiratory conditions across the life course?; (ii) what changes would need to be made at the health system, and at which level, to incorporate the evidence on interventions coming out of RESPIRE projects, what would these cost, and who would pay for these?; (iii) what is the role of donors and international organisations in each of these countries, and what evidence are they demanding in order to tailor their financing packages and mechanisms towards the interventions highlighted by RESPIRE. These issues will be very important to elucidate in detail so that study findings can be properly interpreted and their applicability in other South Asian settings considered for intervention scale-up.

### Platform II: Capacity and capability development

There is a need to develop all levels of research capacity in partner LMICs, ranging from introductory courses in research methods to post-graduate training and courses aimed at executive leaders.

#### MOOCs and Masters

RESPIRE builds on a track-record in The University of Edinburgh of delivering research training opportunities tailored to different needs and contexts. This includes running MOOCs, face-to-face and distance learning, certificate and masters level courses, and training international PhD students from LMICs. RESPIRE aims to develop specialist MOOCs in health services research and respiratory disorders to foster a peer-to-peer learning community. Through an internal scholarship programme, which is a part of its Commonwealth Commission commitments, RESPIRE can provide a limited number of places in the distance-learning Masters courses, eg, on Clinical Trials, Master of Family Medicine, Infectious Diseases, Global eHealth and eMaster of Public Health. Students are selected through a competitive process in each of the partner countries, which includes assessment of the alignment of dissertation project with RESPIRE priorities.

#### PhDs

RESPIRE is supporting PhD candidates from the four partner countries at The University of Edinburgh, whilst enabling them to carry out their primary research activities in their home countries. The students were selected through a competitive process to join the University’s existing cohort of international PhD students. Edinburgh's Global Health Academy (GHA) provides postgraduate students and visiting fellows with links to a range of teaching resources and opportunities, linking them to the GHA community of practice, which includes a wide alumni network of graduates living in LMICs. In the first RESPIRE-funded PhD student cohort, 10 PhD students commenced their studies from 1^st^ April 2018, following the induction week from 26^th^-30^th^ March 2018 to prepare them for their PhD studies. Six of the PhDs are nested within RESPIRE projects, whilst four are stand-alone projects, closely aligned to the RESPIRE research priorities. Each PhD student is supervised by at least two Edinburgh-based supervisors and at least one partner country-based supervisor. They all investigate research questions relating to one of the two programmes of the RESPIRE Unit.

Topics approved for the first cohort of RESPIRE PhD students include development and pilot testing of an intervention on lung cancer and chronic respiratory disease in a rural community, assessing the feasibility of using a well-established teleconsultation facility (Micro-Health Centre -MHC) in management of COPD and asthma in a remote rural area, studying care-seeking practices of and barriers to care-seeking for pneumonia in children aged less than 5 years in rural areas, studying culturally tailored school-based interventions for childhood asthma, developing and piloting an ICT-based intervention for adult asthma with limited health literacy to improve asthma self-management, implementation research on introducing pulse oximetry with routine Integrated Management of Childhood Illness (IMCI) services at health facilities, enhancing access to pulmonary rehabilitation through implementation research, exploring long-term effects of RSV infection in early childhood, community use of digital auscultation to improve diagnosis of paediatric pneumonia in rural areas, and mHealth for improving care.

#### Research leadership and in-country training

RESPIRE will provide executive research leadership training, a bespoke programme aimed at respiratory research leaders in partner LMICs with a view to networking regionally and with UK and international investigators. Training is focused on developing underpinning research infrastructures and applied respiratory research capacity in their countries. This capacity building includes use of decision-making tools that were developed at The University of Edinburgh (eg, the Child Health and Nutrition Research Initiative (CHNRI) method [14-16]) in collaboration with international agencies. Research leaders are taught how to apply these tools using the data platforms in their own country and how to communicate the identified priorities to policymakers. RESPIRE will also provide focussed ‘Research Schools’ to address specific research training needs prioritised by the Partners, in each of their own countries. In this way RESPIRE hopes to reach a broad audience encapsulating clinicians, health care providers, community health workers etc, as well as academics and researchers.

### Platform III: Methodology and data science

This platform is developing partner capacity and provide expertise on methodology and data science required to underpin the long-term delivery of interventions.

In a collaborations with RESPIRE partners, methodological support is provided which is tailored to achieve country and context-specific realisation of the short-, medium- and long-term goals of RESPIRE. This includes methodological support that addresses the specific needs of the individual projects – in terms of design, conduct, analysis and reporting; and including input from statisticians, epidemiologists, health economists, qualitative researchers, health psychologists, and patient experts. The Unit is developing a Methodology Academy which includes methodologists who are involved in RESPIRE projects, from Edinburgh, Bangladesh, India, Malaysia, and Pakistan. Importantly, it is not restricted to just those funded within RESPIRE, so the Academy will encourage participation and input from a wide range of methodologists who share the goals of RESPIRE across the globe. To facilitate this RESPIRE is also developing a Methodology Forum. This will be the interactive web-based platform that will enable delivery of all the methodological interaction required to support the RESPIRE projects and the RESPIRE Methodology Academy.

The sata science elements are more concerned with building solutions across the RESPIRE regions and Programmes, specifically establishing the requirements for efficient and large scale projects which in general will re-use existing data. This includes specifying arrangements for data sharing and the safe and efficient processing of routine data. First it must be established what relevant infrastructure and staff resource exists, in respect of data science focussed ambitions (eg, using routine local or national data to better design randomised trials; using routine data for trial outcomes; and using routine data for long term follow-up; and so on) and hence assessing what additional infrastructure and training is required to realise such data science focussed projects in a sustainable way. The overall aim is then to leverage the RESPIRE projects to create and sustain data science-based solutions to design and deliver efficient projects (including randomised controlled trials) that make a worthwhile difference quickly to the local people. The Unit will address these issues in such a way that the improved infrastructure and increased staff capacity and knowledge gained can readily be applied to a wide range of non-respiratory conditions ie, beyond the scope of RESPIRE.

## PROGRESS TO-DATE

Topics for research projects have been identified by the RESPIRE partners in LMICs, who are taking the lead in developing detailed research proposals with inputs from RESPIRE leadership and the rest of the Programme investigators. Experts across the partner countries have debated and agreed the research priorities and the respiratory questions to focus their efforts on. A CHNRI research prioritisation exercise was also carried out to inform the prioritisation of funding [16]. To date, RESPIRE has developed two-rounds of proposals which have been externally peer-reviewed, and assessed against the CHNRI scores, before being recommended for funding after open review at the RESPIRE UMC. The results of the CHNRI priority setting exercise are presented in a separate paper in this issue [16].

While some projects involve issues that are locally relevant, others have the potential to be conducted across multiple centres in more than one RESPIRE country. Current RESPIRE research projects are: (i) conducting implementation research on introducing pulse oximetry within routine IMCI services at first-level health facilities in Bangladesh; (ii) assessing feasibility and acceptability followed by effectiveness of bubble continuous positive airway pressure (bCPAP) for treatment of Bangladeshi children with severe pneumonia; (iii) studying consequence of RSV infection of young infants in childhood – follow-up of children in the ANISA cohort; (iv) testing an innovative approach to improving pneumonia perception following exploration of under-five pneumonia and asthma in Pakistan; (v) RESPIRE chronic respiratory disease prevalence study (4CCORD); (vi) a feasibility study of prevention, detection and treatment of adult lung disease in a poor, rural population in Tamil Nadu; (vii) Developing and evaluating interventions at patient and practice level to improve asthma care; and (viii) The Malaysian asthma Hajj project.

Seven other projects have been launched which entail a range of research infrastructure developments. These projects are: (i) community use of digital auscultation to improve diagnosis of paediatric pneumonia in Sylhet, Bangladesh; (ii) construction of a computational framework to automatically interpret chest x-rays and diagnose pneumonia in Bangladesh; (iii) documentation of pneumonia case management practices in selected communities in Pakistan; (iv) exploration of pneumonia related policy formation and implementation in Pakistan; (v) development of spirometry predictive values for Indian population; (vi) assessment of the Accredited Social Health Activist (ASHA) programme’s workload and its determinants in India; (vii) exploring the provision of supportive/palliative care for patients with very severe COPD in Malaysia.

## LOOKING AHEAD: A CALL FOR OTHER GROUPS TO JOIN THE RESPIRE COLLABORATION

RESPIRE represents a substantial investment by NIHR which it is hoped will catalyse work within the initial 4 partner countries and through so doing unlock increased investments from other agencies and donors and national sources so that RESPIRE’s wider aims and objectives can be achieved. Equally importantly, RESPIRE will need to build a much larger consortium of international partners if the Unit is to succeed in demonstrating a sizeable impact on the global burden of respiratory morbidity and mortality. As an initial step towards this RESPIRE hosted a meeting in Edinburgh in 2018 of UK-based research projects (many of which are supported by NIHR as Global Health Research Units and Groups) which have a focus on global respiratory problems. As well as RESPIRE, representatives attended from the: NIHR Global Health Research Unit on Lung Health and TB in Africa (IMPALA), NIHR Global Health Research Unit on Mucosal Pathogens (MPRU), NIHR Global Health Research Group on Social Policy and Health Inequalities, NIHR Global Health Research Group on Global COPD in Primary Care (Breathe Well), NIHR Global Health Research Group on Achieving Control of Asthma in Children In Africa (ACACIA), NIHR Global Health Research Group on Addressing Smokeless Tobacco and building Research capacity in south Asia (ASTRA); and the CGRF-funded: Lung health in Africa across the life course, A mathematical modeling framework for tuberculosis burden estimation and economic evaluation of pharmaceutical interventions, and the Tobacco control capacity programme. Not able to attend the initial meeting, but also members of the group are: the NIHR Global Health Research Group on Sepsis, NIHR Global Health Research Group on Respiratory Rehabilitation (Global RECHARGE) and the NIHR Global Health Research Group on Asthma attacks, causes and prevention study in urban Latin America. This group plans to meet regularly so that efforts to tackle global respiratory health challenges can be coordinated and synergised. Additional groups from high-, middle- and low-resource settings alike, are invited to join this ambitious effort and work with RESPIRE to build a true global collaboration that will address the problem of global respiratory illnesses.

## References

[1] GBD 2017 DALYs and HALE Collaborators (2018). Global, regional, and national disability-adjusted life-years (DALYs) for 359 diseases and injuries and healthy life expectancy (HALE) for 195 countries and territories, 1990–2017: a systematic analysis for the Global Burden of Disease Study 2017.. Lancet.

[2] GBD 2017 Disease and Injury Incidence and Prevalence Collaborators (2018). Global, regional, and national incidence, prevalence, and years lived with disability for 354 diseases and injuries for 195 countries and territories, 1990–2017: a systematic analysis for the Global Burden of Disease Study 2017.. Lancet.

[3] GBD 2017 Causes of Death Collaborators (2018). Global, regional, and national age-sex-specific mortality for 282 causes of death in 195 countries and territories, 1980–2017: a systematic analysis for the Global Burden of Disease Study 2017.. Lancet.

[4] GBD 2017 Mortality Collaborators (2018). Global, regional, and national age-sex-specific mortality and life expectancy, 1950–2017: a systematic analysis for the Global Burden of Disease Study 2017.. Lancet.

[5] GBD 2015 Chronic Respiratory Disease Collaborators (2017). Global, regional, and national deaths, prevalence, disability-adjusted life years, and years lived with disability for chronic obstructive pulmonary disease and asthma, 1990-2015: a systematic analysis for the Global Burden of Disease Study 2015.. Lancet Respir Med.

[6] World Health Organisation. Global Health Estimates 2016: Deaths by Cause, Age, Sex, by Country and by Region, 2000-2016. 2018. https://www.who.int/healthinfo/global_burden_disease/estimates/en/. Accessed: 29 November 2018.

[7] https://vizhub.healthdata.org/gbd-compare/. Accessed: 29 November 2018.

[8] Head MG, Fitchett JR, Cooke MK, Wurie FB, Hayward AC, Lipman MC (2014). Investments in respiratory infectious disease research 1997-2010: a systematic analysis of UK funding.. BMJ Open.

[9] Cooksey D. A review of UK health research funding. HM Treasury report 2006. 10.1136/bmj.39059.444120.80 PMC1702444 17170394

[10] Respiratory Syncytial Virus Network. Available: http://www.resvinet.org/. Accessed: 12 November 2018.

[11] Glandon D, Paina L, Alonge O, Peters DH, Bennett S (2017). 10 Best resources for community engagement in implementation research.. Health Policy Plan.

[12] WHO/TDR. 2014. Implementation research toolkit. Geneva, Switzerland: World Health Organization on behalf of the Special Programme for Research and Training in Tropical Diseases. Available: http://www.who.int/tdr/publications/year/2014/9789241506960_workbook_eng.pdf. Accessed: 12 November 2018.

[13] Joint United Nations Programme on HIV/AIDS (UNAIDS). AIDS Vaccine Advocacy Coalition (AVAC). 2011. Good Participatory Practice: Guidelines for biomedical HIV prevention trials, second edition. Available: http://www.avac.org/sites/default/files/resource-files/Good%20Participatory%20Practice%20guidelines_June_2011.pdf. Accessed: 12 November 2018.

[14] Rudan I (2012). Global health research priorities: mobilizing the developing world.. Public Health.

[15] Rudan I, Yoshida S, Chan KY, Sridhar D, Wazny K, Nair H (2017). Setting health research priorities using the CHNRI method: VII. A review of the first 50 applications of the CHNRI method.. J Glob Health.

[16] Rudan I, Agrawal D, Hussein N, Cheong AT, Cunningham S, Dockerell D (2018). Setting research priorities for global respiratory medicine within the National Institute for Health Research (NIHR) Global Health Research Unit in Respiratory Health (RESPIRE).. J Glob Health.

